# Mycetoma: Development of Diagnosis and Treatment

**DOI:** 10.3390/jof8070743

**Published:** 2022-07-19

**Authors:** Xingpei Hao, Marcus Cognetti, Rhonda Burch-Smith, Emerald O’Sullivan Mejia, Gene Mirkin

**Affiliations:** 1 Foot and Ankle Specialists of the Mid-Atlantic, LLC, Rockville, MD 20850, USA; 2 P4Diagnostix, Beltsville, MD 20705, USA; marcus.cognetti@p4dx.com (M.C.); rhonda.burchsmith@p4dx.com (R.B.-S.); emerald.mejia@p4dx.com (E.O.M.)

**Keywords:** antifungal therapy, bacteria, chronic infection, excision, foot, fungi, mycetoma, tropical neglected disease

## Abstract

Mycetoma describes a heterogeneous group of cutaneous and subcutaneous infections caused by either fungi (eumycetomas) or bacteria (actinomycetomas). It is characterized by a triad of clinical symptoms: painless subcutaneous tumor-like swelling, multiple sinuses and fistulas, and discharged grains in pus. This predominantly affects the feet in more than 70% of patients. It is endemic in the “mycetoma belt” regions, including Africa, South America, and South Asia. Autochthonous mycetoma is rare in the United States of America (USA). We recently reported a Latin American immigrant with eumycetoma in the State of Maryland, USA. With millions of immigrants from endemic regions, the true number of mycetomas in the USA is most likely higher than currently recognized. With the aim to raise the awareness of clinicians about mycetoma, especially dermatologists and podiatrists, we update the development of the epidemiology, etiology, clinical presentations, pathogenesis, diagnosis, differential diagnosis, and treatment of mycetoma.

## 1. Introduction

Mycetoma has manifested for many centuries. The clinical features of mycetoma were vividly described as “*Padavalmika*” or “anthill foot” [1], an “ant hill-like foot,” in ancient Indian religious book *Atharva Veda* (knowledge storehouse of *atharvāṇas*, the procedures for everyday life) [2]. It was Gill, in 1842, who first described multiple cases, in detail, in an ancient southern Indian city, Madura (changed to Madurai in 1949), and named it “Madura foot” [3,4]. Carter established fungi as the cause of the disease and used “mycetoma” to describe the tumorous enlargement in 1860 [5]. Based on the causative pathogens, in 1913, Pinoy first divided mycetoma into two basic types: “Actinomycosis” (caused by the *Actinomyces* group) and “True eumycetoma” (caused by true fungi or molds) [6].

It is clear now that mycetoma is a chronic subcutaneous, granulomatous infection caused by either fungi (eumycetoma) or bacteria (actinomycetoma) living in the soil and water. The bacterial and fungal pathogens get into subcutaneous tissue through broken skin barrier, locally incubate and proliferate, and then progress to visible clinical lesions. Typically, mycetoma presents as a triad of painless subcutaneous tumor-like swelling, multiple sinuses and fistulas, and discharged grains in pus [7]. If untreated, the pathogens can spread through muscular fascial spaces, or lymphatics, into muscles and bones, and lead to severe consequences including disfiguration, deformity, disability, amputation, and even death [8,9], as well as causing heavy economic and medical burdens to social communities [3,9,10].

Autochthonous mycetoma is rare in the USA [11]. We recently diagnosed and treated a patient with eumycetoma in the State of Maryland, USA [12]. The patient was an immigrant from Southern America, where mycetoma is frequently encountered in clinical practice, suggesting that the true incidence in the USA may be higher than reported due to the millions of immigrants from endemic regions. It is, therefore, necessary to raise the awareness of clinicians, especially podiatrists and dermatologists, about this neglected tropical disease and update knowledge about the epidemiology, etiology, pathogenesis, clinical presentations, diagnosis, differential diagnosis, and treatment.

## 2. Epidemiology

Mycetoma mainly affects poorer populations in remote rural areas in tropical and subtropical countries at altitudes between 30° North and 15° South, the so-called “mycetoma belt” regions, including Sudan, Somalia, Senegal, Yemen, India, Mexico, and Venezuela [11,13,14]. Other countries with reported mycetoma include Egypt, Mauritania, Kenia, Niger, Nigeria, Ethiopia, Chad, Cameroon, Djibouti, and Somalia in Africa; Colombia, Argentina, and Brazil in Latin America [11]. Most of the patients have not received enough attention, due to their living in remote rural areas that lack enough medical services, including well-trained medical personnel, diagnostic facilities, and treatment options [15]. Mycetoma is, therefore, recognized as a “neglected tropical disease” by the World Health Organization (WHO) [16]. A small number of cases have been reported in other countries, including Israel, Germany, The Netherlands, Turkey, Lebanon, Saudi Arabia, Iran, Philippines, Japan, Sri Lanka, and Thailand [3,11,17,18]. Sporadic cases have been reported in at least 20 states in the USA, including one case we reported in the state of Maryland [12], but more cases were found in the southwestern regions of the USA (Texas and California) [3,19,20].

The global incidences of eumycetoma and actinomycetoma are not equally distributed. Globally, eumycetoma accounts for 40% of mycetoma cases, while actinomycetoma accounts for 60% of them [14]. Eumycetoma is endemic in drier regions, such as Africa, especially in Sudan. These regions are known for their harsh climate with short rainy and long dry seasons and with daily temperatures fluctuating from 45–60 °C to 15–18 °C, which favors the survival of the causative pathogens where fungal infections (*Madurella mycetomatis*) are accountable for more than 70% of mycetoma patients [11]. Actinomycetoma is more prevalent in humid and hot regions, such as South and Central America [3]. In Mexico, 92% of mycetoma cases are actinomycetoma, mainly caused by *Nocardia* (78%) and *Actinomadura madurae* (9%), and only 8% of the cases are eumycetoma [13].

Mycetoma predominantly occurs in men, with the ratio of men to women being 3–5:1. Any age group can be affected, but most of the patients are young adults, between 15 and 40 years of age [3,9], the major labor forces conducting farming, herding, and other outdoor activities; especially those working bare-footed are commonly infected. Patients with poor hygiene, malnutrition, and diabetic disease and those suffering with severe dysfunction of the human immune system are also predisposed to these types of infections [21,22,23,24].

## 3. Etiology

Etiologically, more than 70 species of fungi and bacteria have been reported to be responsible for the development of mycetoma, and the total number of species is likely increasing with the advent of advanced molecular techniques [7,12]. The majority of eumycetomas (90%) more frequently found in Africa are mainly caused by four types of fungi, including *Madurella mycetomatis* (most frequently), *Trematosphaeria grisea* (formerly known as *Madurella grisea)*, *Scedosporium apiospermum* (formerly *Pseudoallescheria boydii),* and *Leptosphaeria senegalensis* [11,25] (Table 1). Actinomycetoma, more endemic in Central and Southern America, commonly occurs from infections with actinomycotic species, including *Actinomadura madurae, Streptomyces somaliensis*, *Actinomadura pelletieri*, *Nocardia brasiliensis,* and *Nocardia asteroids* [3,11,26] (Table 1). All of these pathogens have been detected in soil and water in the endemic regions.

## 4. Pathogenesis

The pathogens, including both bacteria and fungi, found in soil and water enter the human body through broken skin, caused by either thorn pricks, wood splinters, or accidental cuts, and inoculate and proliferate in the subcutaneous tissues, eliciting local and systemic inflammatory reactions from the host [3,31]. In the early stage, a small, painless nodule is formed at the site of injury. With the constant proliferation of bacteria or fungi, the body responds with the accumulation of neutrophils and the release of cytokines and a variety of enzymes; the subcutaneous tissues are then digested, creating a viscous, purulent, or serosanguinous fluid, leading to subcutaneous swelling and increased pressure, eventually breaking down the skin barrier, forming the draining sinuses, and discharging grains with different colors, sizes, and textures (depending on the species of the infecting bacteria or fungi). With the extrusion of the pathogens, the subcutaneous tissues begin a healing process, and the unextruded pathogens (either fungi or bacteria) initiate a new infection, invading uninfected surrounding tissues. The cycle of infection, healing, and reinfection continues without treatment. In the late stage, the infection leads to the causative microorganisms spreading through the fascial planes to the underlying muscles and bones, and consequently, to the destruction and deformity of the osseous and muscular tissues and disfiguration. Mycetoma is accordingly classified as “implantation mycoses” [32].

**Figure 1:**
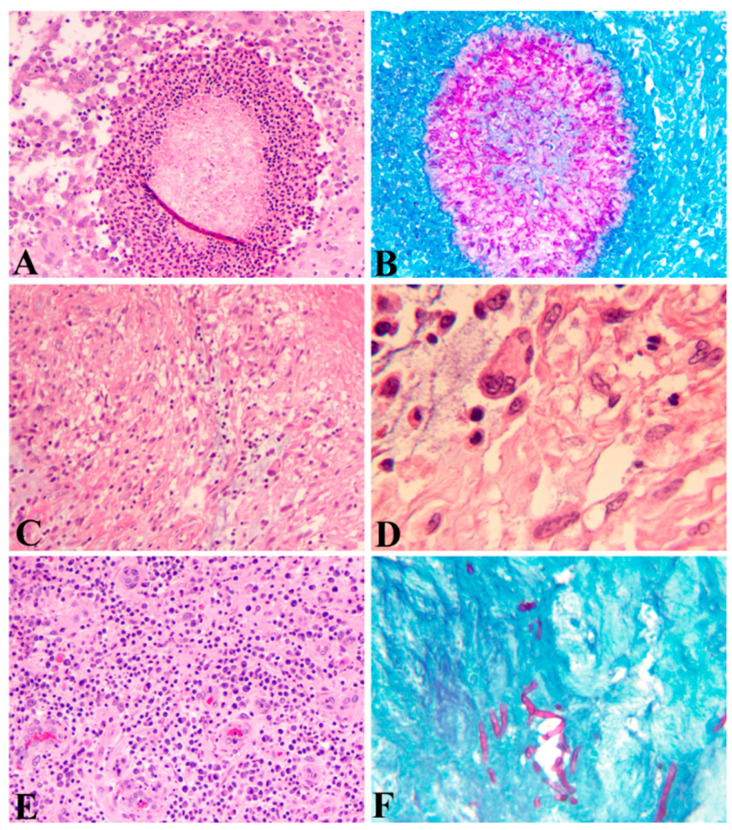
Histopathology of eumycetoma. (**A**) Fungal elements surrounded by a layer of neutrophils and then surrounded by plasma cells, macrophages, lymphocytes, eosinophils, and blood vessels (HE; A; 100 × 1). (**B**) Periodic Acid Schiff (PAS) stain showing fungal elements (100 × 1). (**C**) (HE; 100 × 1) and (**D**) (HE; 400 × 1) Macrophages and Langerhans giant cells predominant in a granuloma. (**E**) Epithelioid granulomas containing lymphocytes, plasma cells, and epithelioid cells, and proliferated blood vessels (HE; 100 × 1). (**F**) PAS stain showing a small number of fungal elements in a granuloma (PAS; 100 × 1).

Histopathologically, the local-host reactions to the infections result in the formation of epithelioid granulomas with either fungi or bacteria inside and with surrounding acute and chronic inflammation. Three types of host-tissue reactions against the mycetoma pathogens have been described that form the basis for histological diagnosis and differential diagnosis. Type I: The fungal elements are surrounded by a layer of neutrophils and then surrounded by plasma cells, macrophages, lymphocytes, neutrophils, eosinophils, and blood vessels, forming a granulomatous tissue, which is further surrounded by a fibrous tissue as the utmost outside layer (Figure 1A), a featured phenomenon called Splendore–Hoeppli. The fungal elements can be stained with PAS (Figure 1B). Type II: Most of the neutrophils are replaced by macrophages, plasma cells, lymphocytes, and multinucleated giant cells (Figure 1C,D). Type III: Formation of epithelioid granulomas, containing Langerhans giant cells and scattered fungal elements among granulomatous tissues (Figure 1E,F) [33,34]. These three types of histological manifestations are not separated events but reflect the different stages of the pathogenesis from acute to chronic infection.

Host systemic reactions to fungi or bacteria include innate and adaptive responses. As with other microorganisms, innate responses, including direct antifungal or antibacterial effector activities and macrophage-mediated phagocytosis, are non-specific, whereas adaptive immune responses, including the activation of specific T cells and the production of pro-inflammatory mediators such as chemokines and cytokines, are specific. Serum Th-1 cytokines include IFN-γ, TNF-α, and IL-2, and Th-2 cytokines such as IL-4, IL-5, IL-6, IL-10, and IL-13 in patients infected with *Madurella mycetomatis* were found to be increased as compared with the control group [23]. Th1 cells and associated cytokines are mainly involved in the cell-mediated immunity and activation of the phagocytes for phagocytosis, whereas the Th2 cells and associated cytokines play important roles in the activation and differentiation of B cells to plasma cells for antibody production as well as the generation of memory B cells [35,36,37]. These reactions correspond to the increased aggregation of neutrophils, lymphocytes, macrophages, and plasma cells surrounding either fungi or bacteria as observed in HE slides (Figure 1A,B).

Increased serum levels of IL8, NO synthase 2 (NOS2), CC Chemokine ligand 5′ (CCL5), and IL10 in mycetoma patients have been linked to genetic alterations in *IL8* (*CXCL8*), *NOS2*, *CCL5*, and *IL10*, as demonstrated by analyses of either single-nucleotide polymorphisms or polymerase-chain-reaction and restriction-fragment-length polymorphisms (PCR-RFLPs) in mycetoma patients [38,39].

Nutritional factors play multiple essential roles in normal cell growth, metabolism, immune function, and inflammatory responses to pathogen invasion. Macronutrients including carbohydrates, proteins, fats, and liquids, as well as micronutrients including amino acids, vitamins, and minerals, are required for the formation and proper function of leukocytes, lymphocytes, and macrophages. Protein and fat deficiencies lead to impaired collagen synthesis, angiogenesis, fibroblast proliferation, reduced production of cytokines, and chemokines including prostaglandins, thromboxane, and leukotrienes in the inflammatory response to invading pathogens. Deficiencies of vitamin A and Zinc result in altered B-cell and T-cell functions and antibody production, decreased cytotoxicity of natural killer cells, impaired phagocytosis of macrophages and neutrophils, and decreased numbers of granulocytes [40,41]. In mycetoma endemic regions, malnutrition in these patients leads to decreased immunity, increased susceptibility to infectious pathogens, including bacteria and fungi, and difficulty in mounting an effective immune response to resolve the infection.

## 5. Clinical Presentation

Despite the different pathogens infected, the clinical manifestations are similar between eumycetoma and actinomycetoma, even though some differences do exist (Table 1).

**Figure 2:**
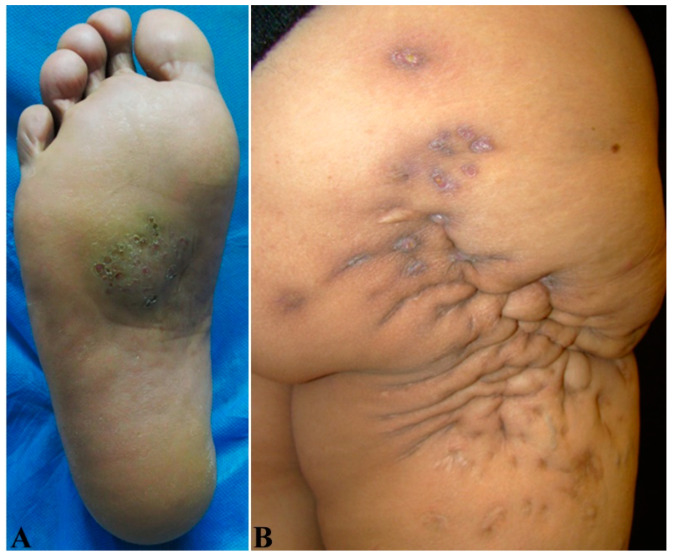
Clinical presentation of actinomycetoma. (**A**) A raised, swelling actinomycetoma with multiple sinuses and yellow discharges on the surface of the middle sole caused by *Nocardia brasiliensis* with an evolution of 7 years in a 43-year-old male patient. (**B**) Multiple, different-staged actinomycetomas on the disfigured gluteus and outer side of the right thigh caused by *Nocardia brasiliensis* with an evolution of 12 years in a 54-year-old female patient. Courtesy of Dr. Jesus Dante Guerra.

**Figure 3:**
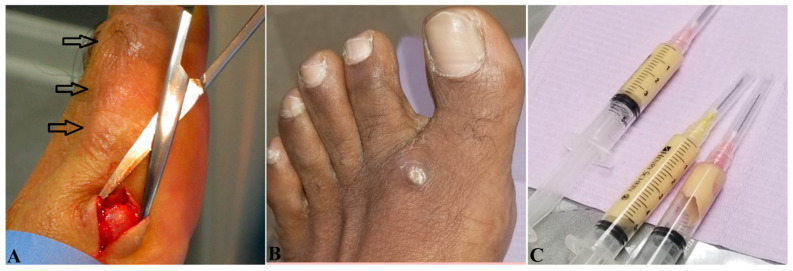
Clinical presentation of eumycetoma variants. (**A**) Multiple nodular eumycetomas without sinuses and discharge on the left dorsal interior foot in a 51-year-old male. (**B**) A 8.0 × 3.0 cm, giant, cystic eumycetoma in the first left intermetatarsal space in a 57-year-old male. (**C**) Thick, yellowish-whitish gelatinous liquid aspirated from (**B**).

Mycetoma is characterized by a typical triad of painless subcutaneous tumor-like swelling; multiple draining sinuses and fistulas; and grainy discharges, which are the colonies of bacteria or fungi, with different colors, sizes, and textures, depending on the species of the causative microorganism(s) (Figure 2A,B). Clinical variants include nodules without sinuses (Figure 3A), cysts (Figure 3B,C) [42], and verrucous plaques [7].

## 6. Diagnosis

**Figure 4:**
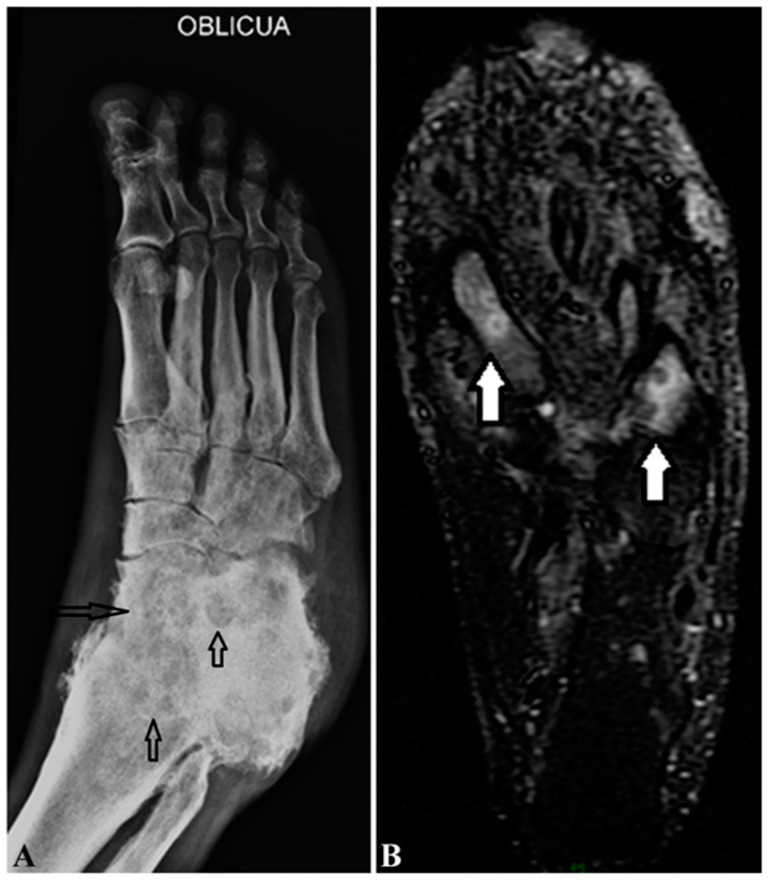
Radiographs of actinomycetoma on a foot. (**A**) Oblique radiograph of actinomycetoma on the right foot revealing multiple, mixed punched-out (lytic and blastic) cavities (black hollow arrows) of the tarsus and obliteration of joint margins. (**B**) Coronal MRI view in STIR sequence of the left foot of a 40-year-old male with actinomycetoma showing at least two lesions in the first and fourth metatarsal bones with “dot in circle” signs (white solid arrows) characterized by a hyperintense circle in T2-weighted sequences with a hypointense center. Courtesy of Dr. Jesus Dante Guerra.

The diagnosis of mycetoma can be made based on the clinical triad of symptoms in endemic regions. However, it is a challenge in non-endemic regions due to the lack of typical clinical presentations and awareness of most clinicians, as we experienced [12]. Therefore, the diagnosis of suspected patients should include a series of tests including a direct microscopic examination of the discharge (Table 1); imaging studies with conventional radiography (Figure 4A), ultrasonography, and MRI (Figure 4B); bacterial and fungal cultures; biopsy for histopathological evaluation with special staining, including PAS (Figure 1) and GMS for fungi, and Gram and AFB for bacteria; and molecular profiling [12] to confirm the diagnosis and identify the species of the causal microorganism.

## 7. Direct Microscopic Examination

Discharged serosanguineous grains obtained from open sinuses, fine-needle aspiration (FNA), punch biopsy or surgical excisional biopsy need to be first grossly examined for their color, size, and consistency as outlined in Table 1 and then smeared on clear slides. One slide is treated with one or several drops of 10% potassium hydroxide (KOH) to digest keratin and other tissue components and then examined directly under a light microscope for fungal spores and hyphae [43]. Another slide needs to be stained with Lugol’s iodine to observe branching filaments for actinomycetes. Direct microscopic examination (DME) provides cost-effective and rapid results at the point of care, and it is especially useful in endemic regions with limited medical resources. It should be borne in mind that discharged grains from open sinuses are commonly non-viable and contaminated, and DME results may lack specificity and accuracy.

## 8. Histopathological Examination

When DME fails to reveal fungal or bacterial elements with discharged grains, deeper tissues are needed for testing. The subcutaneous tissues can be obtained with punch biopsy, local surgical excision, and FNA. FNA specimens can be either imprinted on the slides for further DME or processed for cell blocks and further processed as biopsy specimens for histopathological assessment as described in pathogenesis (Figure 1). Besides making HE slides, unstained slides need to be further stained with PAS (Figure 1B,F) or GMS to visualize the fungal elements, as well as with Gram and Acid-Fast Bacillus (AFB) for bacterial elements (Table 1). Histological evaluation can differentiate the differences between fungal and bacterial infection but is not helpful in identifying the species infected. 

## 9. Grain Culture

Fungal or bacterial cultures can help to identify the causative microorganisms based on the colony’s morphological and biological characteristics, including color, texture, optimal temperature, medium requirement, etc. Antibiotics such as penicillin G, gentamicin sulphate, streptomycin, or chloramphenicol are required for eumycetoma but not for actinomycete culture medium. Modified Sabouraud agar supplemented with 0.5% yeast extract, blood agar, brain–heart infusion (BHI) agar, and Löwenstein agar are the recommended media for eumycetoma [44]. Moreover, a selective medium supplemented with benomyl (1-(butylcarbamoyl) benzimidazol-2-yl methylcarbamate), amphotericin B or Dichlorane Rose Bengal, and chloramphenicol (DRBC) is suitable for the isolation of fungi from the *Scedosporium*/*Pseudallescheria* complex [45,46]. Recommended culture media for actinomycetoma include Sabouraud dextrose agar, thioglycolate broth as liquid medium, Columbia agar, BHI agar, and Schaedler agar (for anaerobic microorganisms) [4,47].

Grain culture is time consuming and technically demanding. The potential contamination and difficult differentiation of the morphology of different types of fungi or bacteria may provide wrong results.

## 10. Molecular Profiling 

With the limitation of the histopathological and culture techniques in identifying definitive species of microorganisms, recently developed molecular methods have greatly facilitated the identification and characterization of the species involved in mycetoma [48,49]. These approaches include multiplex PCR; real-time PCR (RTPCR); restriction-fragment-length polymorphisms (RFLPs); isothermal methods including Recombinase Polymerase Amplification (RPA), Loop-mediated isothermal AMPlification (LAMP), and Rolling Circle Amplification (RCA); and matrix-assisted laser desorption/ionization time-of-flight mass spectrometry (MALDI-TOF MS) [50,51,52]. Each technique has its own features and applications, but the detailed review of the advantages and disadvantages of each technique is out of scope of the current review. With confirmed fungal elements on PAS-stained slides, we extracted DNA from the unstained slides of formalin-fixed, paraffin-embedded tissues; performed polymerase-chain-reaction-based sequencing in a Latin American immigrant; and identified *Cladosporium cladosporioides* as the causal pathogen of eumycetoma [12]. It should be pointed out that fungal elements must be visible on the unstained slides in order to successfully get the fungal DNA extracted, amplified, and sequenced.

## 11. Serodiagnostic Test

Serodiagnostic tests including immunoblots, indirect hemagglutination assays (IHAs), immunodiffusion (ID), counterimmunoelectrophoresis (CIE), and ELISAs were developed to test both actinomycetoma and eumycetoma [53,54,55,56,57]. (1→3)-β-d-Glucan, a cell-wall polysaccharide present in many fungal species, is secreted during the growth and spread of the infection. An increased level of (1→3)-β-d-Glucan was reported in the sera of patients with different fungal infections, including eumycetoma and actinomycetoma [58]. Since (1→3)-β-d-Glucan can be detected in both eumycetoma and actinomycetoma, it is not useful to differentiate between eumycetoma and actinomycetoma. These serodiagnostic tests are still in the laboratory investigational stage, and further work needs to be conducted to apply these tests into clinical practice, including for the diagnosis and monitoring of the efficacy of the treatment.

## 12. Radiography

Radiography, either X-ray, ultrasonography, computed tomography (CT), or magnetic resonance imaging (MRI), is required to reveal a mycetoma’s site, size, extent, bone invasion, and relationship with surrounding tissues and to plan surgical operation. Conventional X-rays are cost-effective and can demonstrate bone cavities (Figure 4A). Eumycotic cavities are usually a few cavities that are ≥10 mm in diameter, while actinomycotic cavities are often multiple but smaller. “Dot-in-circle” signs on the ultrasound sonograph and MRI are characterized by discrete small round hyperintense circles with central hypointense dots in the hypointense matrix of the mass (Figure 4B) [12,59]; the central hypointense dots correspond to the macro- and micro-abscesses, while the hyperintense circles are the fibrous tissues surrounding the abscesses. It is a diagnostic sign of mycetoma in ultrasonography and MRI.

## 13. Differential Diagnosis

Mycetoma needs to be differentiated from infectious and non-infectious diseases. In endemic regions, infectious skin diseases should be considered first, including cutaneous tuberculosis, coccidioidomycosis, chromoblastomycosis, hyalohyphomycosis, sporotrichosis, blastomycosis, dermatophyte pseudomycetomas, and botryomycosis [4]. In non-endemic regions, benign and malignant lesions should be considered first, including fibroma, rheumatoid nodule, keloid, fibrolipoma, dermatofibroma [60], dermatofibrosarcoma protuberans [61,62], Kaposi’s sarcoma [63], malignant melanoma [64], and verrucous carcinoma. Clinical examinations with different diagnostic modalities, including radiography and biopsies, with different laboratory techniques, such as histological evaluation with special staining, fungal and bacterial culture, and molecular profiling can make a final diagnosis. 

## 14. Treatment

Treatments for eumycetoma and actinomycetoma are different [8]. Eumycetoma is treated with antifungal agents in combination with surgical excision, whereas actinomycetoma is treated with antibacterial agents. No specific antifungal medications have been developed for eumycetoma. Currently, recommended agents for eumycetoma include itraconazole (400 mg daily or 200 mg twice daily) [8], voriconazole (400 mg daily) [65,66], posaconazole (800 mg daily) [67], and terbinafine [68], individually or in combination. Ravuconazole was recently shown to be effective against *M. mycetomatis* in an in vitro study [33]. Fosravuconazole was developed to treat onychomycosis [69,70]. It can rapidly convert to ravuconazole in the body once taken orally. A clinical trial to treat eumycetoma with fosravuconazole at Mycetoma Research Center in Khartoum, Sudan, has been conducted since 2017, with promising results. This could add a new potent drug to treat eumycetoma.

Surgical options for eumycetoma include wide local excision, amputation, and debridement [12,71]. Surgical intervention is most suitable for localized lesions, less than 5 cm, or for patients not responding to the aforementioned antifungal therapy. For moderate (>5–10 cm), giant (>10 cm), or multiple lesions involving the bones, itraconazole is initially given for six months to arrest the fungal growth, which reduces the size of the mass(es) and promotes the proliferation of fibroblasts to encapsulate granulomatous tissues. This allows for the surgical excision of the then enclosed lesion, which avoids extensive or multiple surgical procedures [72]. Itraconazole should be continuously given another six months or longer depending on the treatment efficacy. The clinical outcome is not optimistic. The recurrent rate reached 27.2% in an analysis of 1013 patients receiving a combined therapy of itraconazole and surgery [73].

In non-endemic regions such as in North America, especially with atypical, undiagnosed masses, surgical excision is the first choice to not only remove the lesions (Figure 3), but also facilitate the diagnosis, identify the precise species, and initiate the best antifungal therapy, as we did [12].

Actinomycetoma is usually treated with antibacterial agents. Several medications including amikacin, dapsone (diaminodiphenyl sulphone), trimethoprim-sulphamethoxazole (T-S), and streptomycin sulphate are used in different combinations to treat actinomycetoma, depending on the infecting species. *Actinomadura madurae* are treated with streptomycin sulphate and dapsone; *Nocardia spp.* with T-S and dapsone; and in *Streptomyces somaliensis* and *Actimomyces pelletieri* with T-S. Amikacin should be added to T-S and dapsone to treat severe actinomycetoma [4,47]. The current recommended regimen for actinomycetoma is a combined therapy of amikacin and T-S in a five-week cycle. The patients are treated with amikacin sulphate 15 mg/kg/day intramuscularly (divided into two daily doses) for 3 weeks and simultaneously with T-S 8/40 mg/kg/day orally for 5 weeks, up to four cycles, depending on the patient’s response. This treatment leads to 90% cure results [74]. Audiometry and creatinine clearance should be conducted before and after each cycle of amikacin sulphate treatment to monitor ototoxicity and nephrotoxicity.

Other antibiotics, including carbapenems such as imipenem and meropenem, amoxicillin-clavulanic acid, clindamycin, and quinolone, are used in resistant cases [47]. Surgical excision in combination with medical treatment is needed to treat recalcitrant actinomycetoma [75]. The durations of treatment for both eumycetoma and actinomycetoma are usually one–two years for resolution.

## 15. Conclusions

Even though autochthonous mycetoma is rare in the USA, it is necessary to raise awareness about its presence due to the large population of immigrants from endemic regions. The accurate diagnosis of mycetoma requires a detailed clinical history, including immigration status and travel history to endemic regions; a thorough physical examination; imaging studies including X-ray, ultrasound, and MRI; analyses of discharge or aspiration with visual observation; direct microscopic examination; mycological and bacterial culture; molecular sequencing; and histopathological evaluation with special staining. Mycetoma needs to be differentiated from other infectious conditions and non-infectious benign and malignant lesions. Treatment options include long-term antifungal (for eumycetoma) or antibacterial (for actinomycetoma) therapy or a combination of local surgical excision and antifungal and antibacterial treatment for severe cases.

## Figures and Tables

**Table 1:** Characteristics of the major microorganisms and clinical features of eumycetoma and actinomycetoma.

	Eumycetoma	Actinomycetoma
Pathogen	Fungi	Bacteria
Pathogenesis	Slow	Rapid
Gross appearance	Single or multiple nodules with clear margin with rare or a few sinuses (Figure 3A,B)	Diffuse lesions without clear margin and with multiple sinuses and discharge of grains (Figure 2A,B)
Body region involved	The majority on the foot (70%; Figure 2A and Figure 3A,B) and hand (10%) [15]; the remaining in other parts of the body	Most on the foot (60%), followed by the trunk (back and chest), arms, forearms, legs, knees, thighs (Figure 2B), hands, shoulders, and abdominal wall [27]
Colors of grains [28]	Black: *Madurella mycetomatis*, *Trematosphaeria grisea* (formerly *Madurella* *grisea)*, *Leptospheria senegalensis*, *Leptosphaeria thompkinsii*, *Exophiala jeanselmei*, *Medicopsis romeroi* (formerly *P.romeroi*), *Curvularia lunata,* *Phytophthora parasitica*, *Plenodomus avramii*, *Corynespora cassiicola*, *Phialophora verrucosa*, *Pseudochaetosphaeronema larense* White: *Neotestudina rosatii*, *Acremonium falciforme*, *Acremonium kiliense*, *Acremonium recifei*, *Cylindrocarpon cyanescens*, *Cylindrocarpon destructans*, *Scedosporium apiospermum* (formerly *Pseudallescheria boydii)*, *Fusarium oxysoprum*, *Fusarium solani*, *Fusarium moniliforme*, *Cladosporium cladosporioides*, *Polycytella hominis* White or pale: *Aspergillus nidulans* Green or pale: *Aspergillus flavus*	White–yellow or pink: *Actinomadura madurae* Yellow–brown: *Streptomyces somaliensis* Red: *Actinomadura pellitieri* White–yellow: *Actinomyces israelii*, *Nocardia caviae*, *Nocardia farcinica* White: *Nocardia asteroides*, *Nocardia brasiliensis*, *Nocardia transvalensis* Cream: *Nocardia dassonvillei*
Grain morphology	*Madurella mycetomatis:* Large granules (up to 1–2 mm or more) with interlacing hyphae embedded in interstitial brownish matrix; hyphae at periphery arranged radially with numerous chlamydospores *Scedosporium apiospermum* (formerly *Pseudallescheria boydii):* Eosinophilic, lighter in the center; numerous vesicles or swollen hyphae; peripheral eosinophilic fringe	*Actinomadura madurae:* Large (1–5 mm) and multilobulate; peripherally basophilic and centrally eosinophilic or pale-stained; filaments grow from the peripheral zone *Streptomyces somaliensis:* Large 0.5–2 mm or more) with dense thin filaments; often homogenously stained; transverse fracture lines *Actinomyces israelii:* Small grains (approximately 1 mm); central purple zone; loose clumps of filaments; Gram-positive delicate branching filaments breaking up into bacillary and coccal forms; Gram-negative amorphous matrix
Direct microscopy	KOH mount: Fungal hyphae and spores	Lugol’s iodine stain: filaments with a width of 0.5–1 μm
Histospecial staining	Periodic Acid–Schiff (PAS) (Figure 1B,F), Gomori Methanamine Silver (GMS) 2–5 µm wide hyphae	Gram, Acid-Fast Bacillus (AFB) *Actinomadura* grains are Gram positive, with 0.5–1 µm wide branching filaments and are AFB negative *Nocardia* species are Gram positive, and nearly all are weakly acid fast HE: the small grains of *Nocardia* are eosinophilic with a blue center and pink filaments
Bone invasion	Rare or involved after a long time	Rapid
Radiograph	Normal density and structure of the bone if not involved, “punched out” sign on the bone if involved [29]	“punched out” sign on the bones if involved (Figure 4A) [29]
Magnetic Resonance Imaging (MRI) and ultrasonography	“Dot in Circle” [12,29,30] Discrete small round hyperintense circles with central hypointense dots in the hypointense matrix of mass	“Dot in Circle” [29,30] Multiple small round hyperintense circles with central hypointense dots in the hypointense matrix of mass (Figure 4B)
Treatment	Surgical excision plus antifungal therapy	Antibiotics

## Data Availability

No supporting data available.
